# Simultaneously Estimating Process Variation Effect, Work Function Fluctuation, and Random Dopant Fluctuation of Gate-All-Around Silicon Nanosheet Complementary Field-Effect Transistors

**DOI:** 10.3390/nano15171306

**Published:** 2025-08-24

**Authors:** Sekhar Reddy Kola, Yiming Li

**Affiliations:** 1 Parallel and Scientific Computing Laboratory, College of Electrical and Computer Engineering, National Yang Ming Chiao Tung University, 1001 Ta-Hsueh Rd., Hsinchu 300093, Taiwan; 2 Institute of Communications Engineering, College of Electrical and Computer Engineering, National Yang Ming Chiao Tung University, 1001 Ta-Hsueh Rd., Hsinchu 300093, Taiwan; 3 Institute of Biomedical Engineering, College of Electrical and Computer Engineering, National Yang Ming Chiao Tung University, 1001 Ta-Hsueh Rd., Hsinchu 300093, Taiwan; 4 Department of Electronics and Electrical Engineering, College of Electrical and Computer Engineering, National Yang Ming Chiao Tung University, 1001 Ta-Hsueh Rd., Hsinchu 300093, Taiwan; 5 Department of Microelectronics, College of Electrical and Computer Engineering, National Yang Ming Chiao Tung University, 1001 Ta-Hsueh Rd., Hsinchu 300093, Taiwan; 6 Institute of Pioneer Semiconductor Innovation, Industry Academia Innovation School, National Yang Ming Chiao Tung University, 1001 Ta-Hsueh Rd., Hsinchu 300093, Taiwan; 7 Institute of Artificial Intelligence Innovation, Industry Academia Innovation School, National Yang Ming Chiao Tung University, 1001 Ta-Hsueh Rd., Hsinchu 300093, Taiwan

**Keywords:** gate-all-around, nanosheet, vertically stacked, process variation effect, work function fluctuation, random dopant fluctuation, interface trap fluctuation, short-channel effect, current densities, gate capacitances, 3D device simulation

## Abstract

We systematically investigate the combined impact of process variation effects (PVEs), metal gate work function fluctuation (WKF), and random dopant fluctuation (RDF) on the key electrical characteristics of sub-1-nm technology node gate-all-around silicon nanosheet complementary field-effect transistors (GAA Si NS CFETs). Through comprehensive statistical analysis, we reveal that the interplay of these intrinsic and extrinsic sources of variability induces significant fluctuations in the off-state leakage current across both N-/P-FETs in GAA Si NS CFETs. The sensitivity to process-induced variability is found to be particularly pronounced in the P-FETs, primarily due to the enhanced parasitic conduction associated with the bottom nanosheet channel. Given the correlated nature of PVE, WKF, and RDF factors, the statistical sum (RSD) of the fluctuation for each factor is overestimated by less than 50% compared with the simultaneous fluctuations of PVE, WKF, and RDF factors. Furthermore, although the static power dissipation remains relatively small compared to dynamic and short-circuit power components, it exhibits the largest relative fluctuation (approximately 82.1%), posing critical challenges for low-power circuit applications. These findings provide valuable insights into the variability-aware design and optimization of GAA NS CFET device fabrication processes, as well as the development of robust and reliable CFET-based integrated circuits for next-generation technology nodes.

## 1. Introduction

Due to the significant occurrence of short-channel effect (SCE) and the diminished gate controllability, the miniaturization of devices has resulted in notable leakage currents [1,2]. To address this challenge, the semiconductor industry has turned its attention to gate-all-around (GAA) devices, such as nanowire (NW) and nanosheet (NS) metal–oxide–semiconductor field-effect transistors (MOSFETs) [3,4,5,6,7,8,9]. These devices hold a special place due to their capacity to reduce supply voltages, maintain effective gate control, and deliver high performance for advanced technology nodes [10,11,12,13,14,15]. Currently, GAA NS MOSFETs are actively being developed as a viable production method for logic devices in technology nodes under 2 nm [16,17,18,19,20,21]. Nevertheless, these devices face inherent physical limitations as they scale down due to the intricacies of device fabrication [1,22,23,24]. Recent advancements in emerging very-large-scale integration (VLSI) technologies have introduced the concept of GAA NS complementary field-effect transistors (CFETs) [25,26,27,28,29]. Consequently, CFETs advance this concept by enabling the stacking of complementary pairs of transistors on top of each other, effectively incorporating two transistors in one fin. This distinctive innovation involves placing P-FET at the bottom of the fin and N-FET at the top, setting CFETs apart from GAA NS MOSFET [30,31]. CFETs exploit the superior electrostatic control of the GAA NS architecture, making them promising candidates for sub 1 nm technology nodes [30,32,33,34,35,36,37,38]. However, their fabrication process is complex and less repeatable, especially during channel etching and gate metal deposition [1,39,40,41]. CEFTs have garnered significant attention owing to their efficient use of active area through the vertical stacking of both N- and P-FETs [42]. Nevertheless, as nodes shrink below 2 nm, managing the fabrication process becomes progressively challenging, giving rise to various sources of fluctuation attributed to process variations. These variations pose a significant impact on chip yields by introducing fluctuations in crucial device parameters such as threshold voltage (*Vth*) and off-state current (*I_off_*). In parallel, process variation effects (PVEs) and intrinsic parameter fluctuation (IPF), including work function fluctuation (WKF) and random dopant fluctuation (RDF), are critical factors in the design of complementary metal–oxide–semiconductor (CMOS) devices [43,44,45,46,47], particularly in the context of emerging CFET devices [48]. Recent research has explored PVEs and IPF in planar MOSFETs, FinFETs, GAA NW, and NS MOSFETs [49,50,51,52,53,54]. However, in aggressively scaled devices, even minor systematic process variations can significantly affect device performance and reliability. As such, they warrant careful consideration and cannot be neglected. These variations pose a major challenge to continued technology node scaling, impacting critical parameters such as short-channel effects (SCEs), timing characteristics, and power consumption. For instance, Yang et al. [22] investigated two specific sources of process variation—line edge roughness and gate edge roughness—individually in the context of GAA Si NS CFETs. Notably, the same authors reported that, by individually examining each factor, WKF results in the largest variation in characteristics, assuming variations in the sizes of metal grains from 3 to 7 nm [22]. Moreover, the GAA NS CFET architecture exhibits resilience against various sources of process variations [48,55], a critical consideration in modern semiconductor manufacturing. By mitigating the impact of factors such as PVEs, WKF, RDF, and ITF, GAA Si NS CFETs promise more robust and reliable device performance. The WKF has traditionally posed a significant challenge in the variability of FinFETs and GAA NW MOSFETs [56,57]. However, in the realm of GAA NS MOSFETs, a distinctive approach involving the deposition of gate metal around the NS channel using highly conformal techniques, such as atomic layer deposition (ALD), creates an amorphous metal grain in TiN, distinct from physical vapor deposition (PVD) [46,58]. This strategic approach effectively mitigates WKF concerns. Even in the presence of some grain granularity, the relative sizes of grains remain diminutive due to the enlarged gate metal area. This key distinction sets GAA NS MOSFETs apart from FinFETs and GAA NW MOSFETs, thereby reducing the significance of WKF variations [59]. The RDF is a phenomenon in semiconductor devices caused by the random distribution of dopant atoms within the device channel, as well as source-drain extension penetration into the channel dopant [32,60]. In the context of GAA Si NS CFETs, RDF plays a crucial role due to the reduced dimensions of these devices. As the gate length scales down, the discrete nature of dopant atoms becomes more pronounced, resulting in pronounced RDF effects. Understanding and mitigating RDF is vital for ensuring the reliability and predictability of GAA Si NS CFETs in advanced semiconductor technologies. Several studies have investigated the impact of RDF on various types of semiconductor devices. In traditional planar MOSFETs, FinFETs, and GAA NW MOSFETs, RDF has been identified as a source of variability affecting device performance [18,61,62]. However, its significance becomes even more critical in the case of GAA Si NS CFETs, where the inherent challenges of reduced dimensions exacerbate RDF effects. Recent studies on RDF in semiconductor devices have highlighted the need for comprehensive modeling and simulation to understand the intricate interactions between dopant atoms and device characteristics. Various strategies, such as statistical modeling and advanced fabrication techniques, have been explored to mitigate the impact of RDF in different transistor architectures. In the context of GAA Si NS CFETs, limited research has been conducted on the specific influence of RDF, making it an essential area for further exploration. On the other hand, the ITF in semiconductor devices pertains to the variability in the density of interface traps found at the interface between the channel and gate oxide. These interface traps represent localized energy states capable of trapping charge carriers, thereby influencing the performance and reliability of semiconductor devices. The density of these traps can fluctuate due to various factors, including manufacturing processes, material defects, and environmental conditions. The presence of ITF may lead to undesirable consequences such as increased leakage currents, diminished carrier mobility, and alterations in electrical characteristics. In the realm of nanoscale devices, the intricate relationship between surface roughness and interface trap density plays a pivotal role in shaping the overall performance and reliability of electronic components. Due to the strong gate controllability and increased gate–channel interface area in nanosheet devices, the impact of the ITF is significantly reduced and becomes nearly negligible. Therefore, in this study of IPF, we exclude ITF and focus only on WKF and RDF. However, the impact of PVE and IPF factors on electrical characteristics and power fluctuations of CFETs and CFET-based circuits has not received sufficient attention [35,63]. This study expands upon our prior work by offering a more comprehensive examination of the combined impact of PVE and IPF factors on DC, AC, and power fluctuations [5,10,32]. In addition, analyzing variability factors individually and summing their statistical effects often overestimates the total impact compared to a simultaneous variation approach. This is due to the complex interactions and correlations among the factors, which are captured only when they are considered collectively. In this study, we, for the first time, conduct a statistical analysis of device characteristics and power fluctuations of CFET and its circuits, considering the combined influence of PVE and IPF factors. Understanding the interplay of these factors is essential for the design and optimization of GAA Si NS CFETs, as they collectively impact device performance, power consumption, and reliability. Comprehensive exploration and mitigation strategies for this fluctuation are crucial to unlock the full potential of GAA Si NS CFETs in advancing semiconductor technology. In this context, understanding the intricacies of the GAA Si NS CFETs, their design principles, and the underlying physics becomes imperative. This research aims to delve into the comprehensive exploration of GAA Si NS CFETs, shedding light on their unique attributes and paving the way for their integration into the next generation of semiconductor technologies. In addition, the research will contribute to a better understanding of the challenges and opportunities associated with CFET devices in emerging VLSI technologies [30,64]. In this research, we conduct a comprehensive computational analysis of the combined PVE and IPF factors on DC, AC, and transient properties, as well as power consumption, of GAA Si NS CFETs. We employ a unified statistical three-dimensional (3-D) device simulation approach to explore this combined effect. To ensure the utmost accuracy of our simulations, we experimentally validate our methodology. Our investigation focuses on three major PVE factors that can potentially affect device characteristics, namely NS thickness (*T_NS_*), NS width (*W_NS_*), and gate length (*L_G_*). Furthermore, since the impact of IPF on device characteristics is comparatively minor due to the significant screening effect induced by the inversion layer, as part of IPF, we incorporate the effect of WKF and RDF [17,65]. We evaluate overall fluctuations induced by PVE and IPF factors by examining the relative standard deviation (RSD), which is the ratio of standard deviation to the mean value (i.e., RSD = (*σ*/*μ*) × 100%) for each figure of merit.

This paper is organized as follows: Section 2 describes the computational device and configuration of our statistical device simulation. The DC, SCEs, AC, transient, and power consumption results are presented and analyzed in Section 3. Section 4 concludes the findings of this work.

## 2. Three-Dimensional Statistical Device Simulation of PVE and IPF Factors

**Figure 1:**
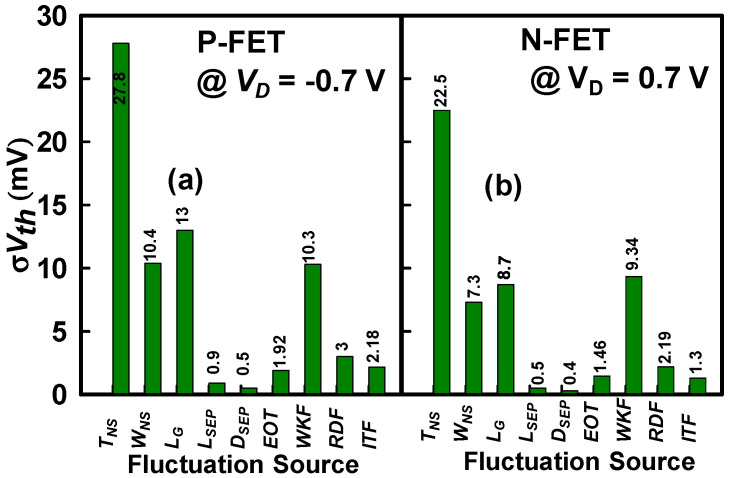
(**a**,**b**) A comparative analysis of the *Vth* variations induced by various random fluctuation sources, including PVE, WKF, RDF, and ITF. The results reveal that the variation in the *Vth* induced by ITF is significantly smaller than that caused by PVE, WKF, and RDF; consequently, the impact of ITF is considered negligible and excluded from further analysis. (**a**) P-FET. (**b**) N-FET.

To validate the vertically stacked GAA Si NS CFET structure, we first calibrated our device simulations against experimentally measured data from an Intel^®^ silicon CFET sample [1], which was reported in our prior work [10]. The calibration was carried out for a gate length of 75 nm, and the validated simulation framework was subsequently scaled down to a gate length of 16 nm. This scaled device serves as the basis for analyzing both PVE and IPF simultaneously. The simulation setup was meticulously tuned to achieve excellent agreement with the measured transfer characteristics. The corresponding calibrated parameters, along with the scaled gate length, are listed in Table 1. For precise and reliable calibration, several critical process and device parameters were systematically optimized. Specifically, the channel doping concentration, source/drain doping profiles, and source/drain extension doping levels were finely adjusted to closely replicate the experimental device behavior. In addition, the effective metal work function within the high-κ metal gate (HKMG) stack was carefully modulated to accurately reproduce the off-state leakage characteristics and the *Vth* [66,67]. Key mobility-related parameters were also refined to enhance the physical accuracy of the simulation; in particular, the high-field carrier mobility was precisely tuned to match the on-state drive current, ensuring accurate modeling of carrier transport under high electric field conditions. The mobility models employed in the simulation framework include the Philips unified mobility model, a surface-orientation-dependent high-field saturation model, a thin-body mobility model, and the Lombardi mobility degradation model for high-κ dielectrics, collectively accounting for phonon scattering, Coulomb scattering, and surface roughness effects at the channel/gate dielectric interface [68,69,70]. To further capture non-equilibrium carrier transport phenomena at nanoscale dimensions, a low-field ballistic mobility model was incorporated to account for quasi-ballistic carrier injection [71]. Moreover, to ensure a comprehensive description of carrier generation–recombination mechanisms, the Hurkx band-to-band tunneling model and the Shockley–Read–Hall recombination model were also implemented within the simulation framework [72]. In addition, the 3-D device simulations were performed by self-consistently solving the quantum-mechanically corrected density gradient (DG) model in conjunction with the classical drift–diffusion (DD) (DD + DG) transport model using the industry-standard Sentaurus TCAD tool [73]. The accuracy of these model equations has been validated against the nonequilibrium Green’s function (NEGF) formalism, ensuring reliable modeling of quantum confinement effects in nanoscale devices [57,74]. To accurately capture carrier transport, the electron effective mass was also adjusted within physically reasonable limits, based on parameters consistent with the adopted transport models [10]. The electron effective mass was adjusted to m* = 0.924 m_0_, where m_0_ denotes the rest mass of a free electron. It is important to note that such modifications to the effective mass significantly impact the carrier mobility model. To preserve model consistency, this adjusted effective mass was applied uniformly throughout the simulation framework. Following the validation of the nominal device simulation for accuracy, we proceeded to investigate the effects of PVE and IPF on the performance of the GAA Si NS CFET. A comparative analysis of various fluctuation sources—PVE, WKF, RDF, and ITF—on the *Vth* variability is presented in Figure 1a,b. Among these factors, variations in the *T_NS_*, *W_NS_*, *L_G_*, WKF, and RDF are identified as having the most pronounced impact on *Vth*. The influence of the remaining factors, such as ITF, is minimal and is therefore neglected in subsequent analyses. Notably, the results confirm that the *Vth* variation induced by ITF is significantly smaller than the contributions of PVE and WKF.

**Figure 2:**
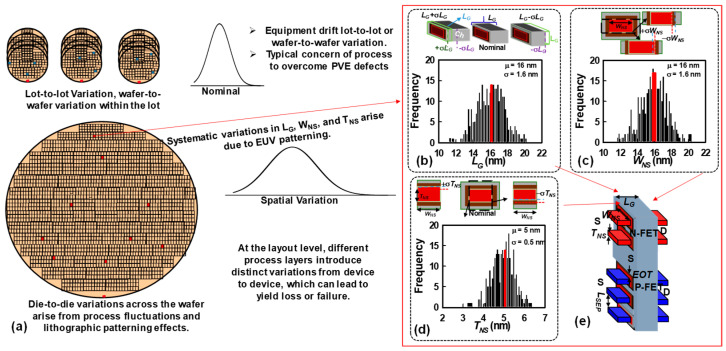
(**a**) Process variations can arise at multiple levels, including lot-to-lot, within-lot, wafer-to-wafer, and even die-to-die across the same wafer. These variations originate from a variety of factors, including lithography limitations, etching non-uniformities, deposition fluctuations, and other process-induced effects. At the layout level, different process layers introduce distinct sources of variability from device to device, which in turn can degrade overall performance, reduce manufacturing yield, or even cause complete functional failure. (**b**–**d**) Overview of the PVE parameters and their implementation in a 3-D GAA Si NS CFET structure, which we also showed in our previous work [10]. The key PVE parameters include (**b**) gate length, (**c**) channel width, and (**d**) channel thickness, each characterized by mean (μ) and standard deviation (σ). (**e**) These parameters are embedded into the 3-D device structure with systematic variations of up to ±10% of their respective μ values. PVE factors are simultaneously incorporated into the simulations along with inter-die IPF sources.

Process variations can manifest at multiple hierarchical levels of semiconductor manufacturing, including lot-to-lot, within-lot, wafer-to-wafer, and even die-to-die across the same wafer. Lot-to-lot and wafer-to-wafer variations often arise from differences in equipment calibration, material properties, or environmental conditions during separate fabrication runs. Within-lot and die-to-die variations, on the other hand, are more strongly linked to local fluctuations in critical process steps such as lithography, etching, and deposition. These include issues such as line-edge roughness, non-uniform photoresist exposure, etch bias variations, or variations in film thickness across different regions of the wafer, as shown in Figure 2a. At the device layout level, additional sources of variability are introduced because each process layer—such as gate oxide growth, channel definition, or contact formation—can deviate slightly from its intended dimensions or material properties. Such deviations accumulate across multiple layers and directly impact key device parameters, including threshold voltage, subthreshold slope, drive current, and leakage current. The compounded effect of these variations not only contributes to significant device-to-device mismatch but can also degrade circuit-level performance metrics such as speed, power consumption, and noise margins. Ultimately, if left unmitigated, these process-induced and layout-dependent variations can lead to reduced manufacturing yield, parametric yield loss, or, in severe cases, complete functional failure of the fabricated circuits. Figure 2e provides a 3-D schematic illustration of the vertically stacked GAA Si NS CFET architecture. This design features two GAA channels for the bottom P-FET, accompanied by an additional parasitic channel, whereas the top N-FET incorporates two GAA channels, carefully engineered to balance the on-state current (*I_on_*) characteristics between the N-FET and P-FET devices. As previously noted, the calibrated simulation parameters—summarized in Table 1—reflect the nominal device characteristics prior to the incorporation of PVE and IPF factors. These calibration methodologies and physical models were consistently applied in subsequent simulations to assess the impact of variability. To thoroughly investigate the effects of PVE and IPF factors, a statistically significant dataset comprising 300 randomly generated samples was created, following a Gaussian distribution, using our own statistical generator [69]. Each sample represents a complete CFET unit, consisting of both an N-FET and a P-FET, and incorporates all random variation factors simultaneously to realistically model the combined impact of PVE and IPF factors on device performance. For the process variability study, three primary geometrical variation components were considered: *T_NS_*, *W_NS_*, and *L_G_*, as depicted in Figure 2b–d, where each PVE parameter is assigned a σ value equal to 10% of its mean value. The numeric range of device parameters varies within the constraints of ±σ, following the guidelines of the 2023 IRDS roadmap [33]. The variations in device parameters were constrained within a range of ±σ, adhering to a Gaussian distribution, as illustrated in Figure 2. Figure 2b–d visually present the distribution profiles of the primary random sources responsible for PVE. Importantly, the simultaneous simulation of both the N-FET and P-FET devices within the GAA Si NS CFET configuration ensures a comprehensive evaluation of variability effects. These random variations were systematically incorporated into the 3-D CFET structure shown in Figure 2e, where fluctuations of up to ±10% relative to their nominal values were introduced. Furthermore, PVE factors were simultaneously integrated with inter-die IPF factors during the simulations to capture the full spectrum of variability influences on the device’s electrical characteristics. Unlike previous studies that examined the impact of each factor independently, we explore both individual and simultaneous effects. This approach allows us to consider the interplay among all factors, capturing their potential synergistic effects or counteracting influences. Notably, our methodology extends beyond previous studies, enhancing our understanding of the complex interactions between these PVE and IFP factors. Additionally, it is worth mentioning that a similar observation regarding the impact of IPF factors on the band profile at the off state has been documented in our earlier work for GAA Si NS MOSFETs [51].

**Figure 3:**
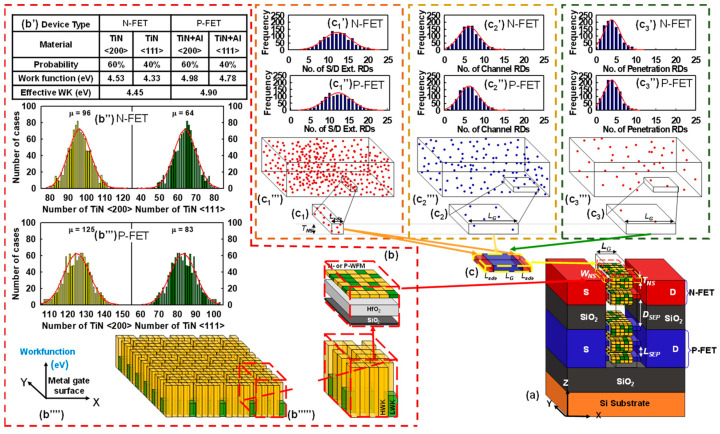
(**a**) A 3-D schematic representation of the GAA Si NS CFET with a HKMG stack, accounting for combined comprehensive random sources, including WKF, RDF, and PVE [32]. (**b**) Each GAA channel’s N-/P-work function metal (WFM) is discretized into small metal grains. (**b’**) The TiN is chosen for N-FET, with two possible orientations, <200> and <111>, occurring with probabilities of 60% and 40%, respectively. The WK values for TiN <200> and <111> are 4.53 eV (HWK) and 4.33 eV (LWK), resulting in an effective WK of 4.45 eV. Conversely, for P-FET, TiN doped with aluminum (Al) is employed. (**b’’**,**b’’’**) The metal grains are generated from a Gaussian distribution within a large plate, and specific distributions are assigned for each case. (**c**) Depicted is the schematic representation of the RDF simulation within the channel region. (**b’’’’**) The alignment of the metal work function with respect to the metal gate surface. (**b’’’’’**) The work functions of HWK and LWK are generated following a Gaussian distribution with parameters (**b’’** and **b’’’**). Subsequently, a small subset is randomly selected for each device. (**c_1_**–**c_1_’’’**) These subsections illustrate the generation of S/D extensions. (**c_2_**–**c_2_’’’**) Channel RDF generation. (**c_3_**–**c_3_’’’**) The penetration RDs are generated based on a Gaussian distribution.

In the context of WKF, it is crucial to note that the work function metal comprises an assigned number of metal grains (MGs), as illustrated in Figure 3b. Each MG of TiN has a size of 2.5 × 2.5 nm^2^ and is of the amorphous type [75,76]. Importantly, the combined fluctuations arising from PVE impact the size of the MGs, which may vary within the range of 0.2–0.4 nm, influenced by variations in PVE parameters. For the N-FET, our simulation employs TiN, which is assigned with probabilities of 60% for <200>-orientation and 40% for <111>-orientation. Furthermore, the work functions of TiN <200> and <111> are 4.53 eV (high work function; HWK) and 4.33 eV (low work function; LWK), respectively, resulting in an effective work function of 4.45 eV. On the other hand, for P-FET, we utilized TiN doped with aluminum (Al). In our simulation, TiN is used as the gate electrode metal for both N-FET and P-FET. However, to achieve the appropriate work function difference required for proper threshold voltage control, we model the P-FET gate using TiN doped with Al. For the N-FET, we use pure TiN with different grain orientations to induce work function variation. The P-FET’s intrinsic work function is not sufficiently high; therefore, Al doping is introduced into TiN to modulate the work function towards values suitable for P-type operation. This approach is consistent with experimental practices, where metal gate tuning via alloying or doping is a common method for achieving the required work function alignment for complementary CMOS devices. The number of HWK and LWK grains follows a Gaussian distribution. For RDF, we randomly generate 6910, 13,538, and 4352 dopants in substantial cuboids comprised of smaller cuboids. These dopants contribute to equivalent doping concentrations of 8 × 10^17^, 2 × 10^19^, and 3.4 × 10^17^ cm^−3^ for the channel, source (S)/drain (D) extension (S_ext_/D_ext_), and penetration from the S/D extensions into the channel (PE), as depicted in Figure 3(c_1_–c_3_). The generation of random dopants in these large cuboids has a Gaussian distribution. These cuboids are subsequently subdivided into 300 smaller cuboids for cases involving fluctuations. On average, each channel contains 11 S_ext_/D_ext_ dopants, seven channel dopants, and four penetration dopants. Statistically generated samples encompassing PVE, WKF, and RDF are factored into a comprehensive set of 3-D quantum-mechanically corrected density gradients, coupled with drift–diffusion transport equations to estimate characteristic fluctuation [77]. We employed similar statistical methodologies to model the concurrent effects of PVE, WKF, and RDF, as illustrated in Figure 2 and Figure 3. A substantial dataset of 300 samples was randomly generated, conforming to a Gaussian distribution, through the simulation program mentioned earlier. This extensive dataset was crucial for a comprehensive evaluation of the combined influence of PVE and IPF on the targeted GAA Si NS CFET devices. To ensure a thorough exploration of the impact of random PVE and IPF factors, we introduced all relevant random factors simultaneously in these simulations.

## 3. Results and Discussion

In this section, we conduct a detailed investigation into the impact of both PVE and IPF factors on the electrical characteristics of vertically stacked GAA Si NS CFETs, analyzing both N-FET and P-FET devices. To systematically evaluate these effects, we examine the variations in key DC characteristics, including *Vth*, off-state leakage current (*I_off_*), *I_on_*, subthreshold swing (*SS*), drain-induced barrier lowering (*DIBL*), and transconductance (*g_m_*). Furthermore, in the context of AC characteristics, we investigate the fluctuations in gate capacitance (*C_G_*), which is sensitive to the changes induced by the combined influence of PVE and IPF factors. Our analysis is further extended into the transient response of the CFET timing domain, evaluating the variations in critical timing parameters such as high-to-low transition time (*T_HL_*), low-to-high transition time (*T_LH_*), rise time (*t_r_*), and fall time (*t_f_*). Additionally, we scrutinize fluctuations in total power dissipation (*P_total_*), encompassing dynamic power (*P_dyn_*), short-circuit power (*P_sc_*), and static power (*P_static_*). Beyond the device-level analysis, we extend our investigation to the circuit level by examining the voltage transfer characteristics (VTCs) of the CFET-based CMOS inverter and conducting a comprehensive evaluation of its noise margins (*NMs*), which are critical for assessing logic-level robustness under variability. Furthermore, we implement and analyze a 6-transistor (6T) static random-access memory (SRAM) cell based on GAA Si NS CFETs, focusing specifically on the degradation in static noise margin (*SNM*) resulting from the PVE and IPF factors [78]. These variability sources introduce significant statistical fluctuations in key transistor parameters such as *Vth*, mobility, and *SS*, which collectively influence read/write stability and data retention in the SRAM cell. The combined impact of PVE and IPF factors manifests as increased dispersion in circuit-level performance metrics, underscoring the necessity of variability-aware design strategies in advanced technology nodes employing 3-D-integrated device architectures such as CFETs.

**Figure 4:**
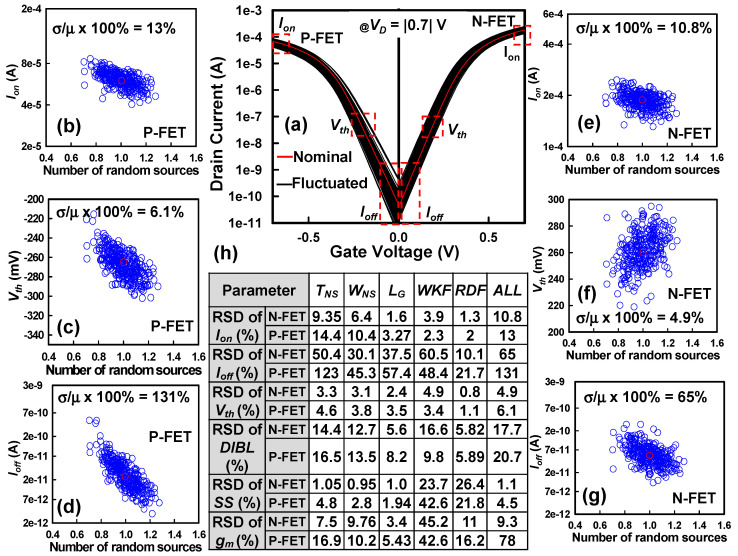
(**a**) The *I_D_*–*V_G_* curves exhibit fluctuations for both N-FETs and P-FETs of CFET devices under saturation drain bias conditions, resulting from the combined impact of PVE and IPF factors. The solid red lines represent the nominal device behavior, simulated without incorporating PVE or IPF, serving as a reference. In addition, all the red circles represent nominal device. (**b**–**d**) The graphs illustrate the variations in *I_on_*, *Vth*, and *I_off_* concerning the combined number of random sources (PVE and IPF factors) for P-FETs, where the *x*-axis (Number of random sources) values are normalized with the mean values of PVE and IPF factors. Likewise, the corresponding graphs (**e**–**g**) depict the variations in *I_on_*, *Vth*, and *I_off_* concerning the combined number of random sources for N-FETs. In all plots, the open red circles represent the nominal device. (**h**) Comparison of the RSD variation of SCE parameters between individual random sources and their combined effect.

Figure 4a illustrates the statistically fluctuated *I_D_*−*V_G_* characteristics for both N-FET and P-FET components of the CFET structure under the influence of PVE and IPF factors. The solid red lines represent the nominal device behavior, simulated without incorporating PVE or IPF, serving as a reference. The results highlight significant variability introduced by the combined stochastic effects, with particularly prominent deviations observed in P-FETs compared to the N-FETs. The pronounced fluctuations in P-FETs are primarily attributed to the presence of a parasitic leakage channel at the device bottom. During the fabrication of GAA NS devices on bulk Si substrates, the nanosheets are released by selectively etching SiGe sacrificial layers. However, simultaneous deposition of the HKMG stack can result in the formation of a parasitic channel at the bottom surface. Unlike the upper GAA channels, which are fully wrapped by the gate dielectric and metal gate, this parasitic channel is only partially controlled—governed mainly by the top and sidewall gates (resembling a FinFET structure)—thus leading to inferior electrostatic control. As a result, the bottom leakage channel substantially contributes to increased variability in P-FET characteristics, particularly influencing the *I_off_* behavior. The *I_off_* is extracted at *V_G_* = 0 V and *V_D_* = |0.7| V. Further analysis of the fluctuation trends reveals that *I_off_* experiences more severe variations in P-FETs compared to N-FETs. This observation motivates a deeper investigation into the sensitivity of SCE parameters and power consumption metrics to the combined influence of PVE and IPF factors. In GAA NS MOSFETs, carrier transport is primarily confined and modulated through the surrounding gate structure, enabling superior electrostatic control compared to planar devices. The extensive GAA configuration effectively suppresses SCEs but also renders device performance highly sensitive to nanoscale process fluctuations. Accordingly, carrier scattering and subthreshold conduction become strongly dependent on variations in the *T_NS_*, *W_NS_*, and *L_G_*—key PVE parameters influencing *I_off_*. Figure 4b–d present the extracted dependence of *I_on_*, *Vth*, and *I_off_* on the number of combined random sources, normalized relative to their nominal values. The *I_on_* is extracted at *V_G_* = *V_D_* = |0.7| V. As depicted in Figure 4b, *I_on_* shows a gradual decline as the number of superimposed variability sources increases, with an RSD of 13% observed for the P-FET. Figure 4c indicates that *Vth* similarly decreases with increasing number of random sources, exhibiting an RSD of 6.1%. Figure 4d highlights the most significant variability in *I_off_*, where P-FETs show dramatic fluctuations, with an RSD reaching 131%. The substantial *I_off_* variation stems from the parasitic leakage exacerbated by variability in physical dimensions and material properties, particularly under the influence of PVE and IPF factors. For N-FETs, Figure 4e–g similarly illustrate trends for *I_on_*, *Vth*, and *I_off_*. Although fluctuations are observed, they are relatively smaller compared to P-FETs, with an RSD of 10.8% for *I_on_*, 4.9% for *Vth*, and 65% for *I_off_*. This difference further underscores the asymmetry introduced by structural and electrostatic differences between N- and P-type devices in CFET architectures. Figure 4h compares the variability induced by individual random sources versus the simultaneous application of PVE and IPF factors (*ALL*). The results clearly indicate that isolated variability factors tend to overestimate total fluctuation levels when statistically summed, compared to comprehensive multi-factor simulations. This highlights the necessity of accounting for complex interaction effects, such as mutual compensation (annihilation) and reinforcement (enhancement), among different PVE and IPF factors. In the context of CFET variability, WKF emerges as the dominant source impacting *Vth*, subsequently influencing the *SS* and *g_m_* characteristics for both N-FETs and P-FETs. WKF-induced variation directly modifies the energy barrier height at the gate–channel interface, significantly affecting carrier injection and tunneling phenomena, especially in the subthreshold regime. Moreover, while RDF is traditionally significant in bulk devices, its impact is substantially mitigated in GAA structures due to the strong electrostatic screening provided by the GAA geometry. This further enhances device immunity to RDF but elevates the relative influence of geometric and interface-related variability sources. Finally, similar trends of IPF-induced off-state band profile modifications have been corroborated in our previous studies on alternative MOSFET architectures, reinforcing the critical role of multi-source variability modeling for next-generation CMOS scaling [61,69].

**Figure 5:**
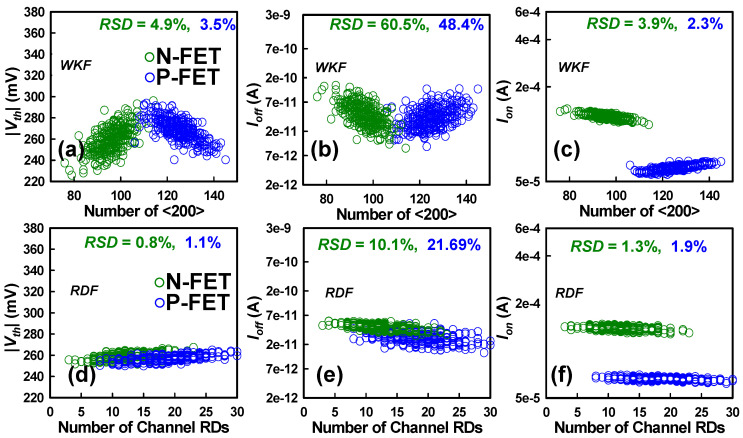
Variations in *Vth*, *I_off_*, and *I_on_* induced by IPF factors, specifically the number of <200> high work function (HWK) metal grains for WKF, and the number of random dopants in the channel for RDF. The analysis includes both N-FET and P-FET devices under a bias condition of |V_D_| = 0.7 V. Subfigures (**a**–**c**) illustrate the WKF-induced variations, while (**d**–**f**) illustrate the RDF-induced variations.

The SCE fluctuations induced by PVE are not shown here, as they have been detailed in our recent study [10]. For comparative analysis of the individual impacts of PVE, WKF, and RDF, we do not present the corresponding I_D_–V_G_ characteristics; instead, the comparison is summarized in tabular form. Figure 5a–c illustrate the SCE variations caused by WKF in both N-FET and P-FET of the CFET device. Notably, the study highlights a significant reduction in fluctuations induced by WKF compared to PVE. The incorporation of amorphous metal grains emerges as a pivotal factor in minimizing characteristic fluctuations. In contrast to crystalline metals with larger grain sizes, amorphous metals effectively reduce grain sizes to less than 3 nm, resulting in an increased number of amorphous metal grains. This surge contributes to a noticeable reduction in characteristic fluctuations. The impact of SCE parameters influenced by WKF is found to be relatively insignificant for both N- and P-FETs when compared to the effects of PVE. Particularly, I_on_ experiences remarkably low fluctuation under the influence of WKF. In Figure 5a–c, the influence of WKF on *Vth*, *I_off_*, and *I_on_* for both N- and P-FETs is depicted. The increasing number of high work function <200> metal grains indicates a pronounced impact of *Vth* and *I_off_*. The increased number of HWK MGs in N-FET leads to an increase in *Vth* and a decrease in *I_off_* due to modification of the energy barrier at the channel interface. A higher WK creates a stronger energy barrier for electrons in the channel, requiring a higher *Vth* to initiate the flow of current. Simultaneously, the increased energy barrier impedes the *I_off_* by restricting electron tunneling through the barrier, resulting in a reduction in *I_off_* in N-FETs. Moreover, in the P-FET, the relationship between the metal WK, *Vth,* and *I_off_* is reversed compared to the N-FET. When the number of HWK MGs increases in P-FETs, the *Vth* tends to decrease and the *I_off_* tends to increase. This is because, in P-FETs, the carriers are holes, and the energy barrier is influenced differently. A HWK in the gate material lowers the barrier for holes, leading to a lower *Vth* and increased *I_off_* in P-FETs. The impact of WKF on both N- and P-FETs is not as pronounced because the primary factor governing *I_on_* is the availability of charge carriers in the channel. In addition, in the *I_on_*, carriers are injected into the channel, and their mobility and concentration are crucial for current flow. The WK of the gate material primarily affects the energy barrier for carriers at the channel interface, influencing *Vth*; however, once the device is in the *I_on_*, carrier concentration and mobility play more significant roles, and the impact of WKF becomes relatively minor in comparison. The RSD of *Vth* stands at 4.9% for N-FET and 3.5% for P-FET. Simultaneously, the increased number of high work function metal grains results in decreased *I_off_* for N-FET and increased *I_off_* for P-FET. In GAA Si NS CFETs, the *I_off_* is significantly influenced by the work function of the gate material, as it plays a pivotal role in controlling carrier flow in the channel region. Both N- and P-FETs exhibit substantial RSD exceeding 49% in *I_off_*. In comparison to PVE, the fluctuations in *I_on_* due to WKF are minimal for both devices in CFET. In addition, Figure 5d–f illustrate that the impact of RDF on the fluctuations in *Vth*, I_off_, and *I_on_* is less pronounced. The presence of increased channel random dopants shows minimal effect on the characteristics fluctuations in *Vth*, *I_off_*, and *I_on_*, resulting in significantly lower variations. The RSD values for *Vth* are 0.8% and 1.1% for N- and P-FETs, respectively, while the RSD values for *I_off_* are 10.1% and 21.5%. For *I_on_*, the RSD values are 1.3% and 1.9%. Among these parameters, *I_off_* exhibits marginal variations. Table 2 presents a comparison of the RSD variation between individual random factors and simultaneous random factors. The statistical *RSD_SUM_* of the RSD variation of the individual random factor is given by the following function:

(1) $${RSD}_{SUM}=\sqrt{\sum {RSD}_{i}^{2}},\;i\in \{T_{NS},W_{NS},L_{G},WKF,\;RDF\}$$

where i is the random factor. The results indicate that the PVE factors have a more dominant influence on the fluctuation of characteristics compared to simultaneous factors. In the context of GAA Si NS CFETs, WKF holds substantial influence over key device parameters, notably the *Vth*. This influence, subsequently, impacts both SS and g_m_ for N- and P-FETs. In addition, it plays a crucial role in determining the energy barrier for carrier injection and tunneling in the sub-threshold region, contributing significantly to variations in SS for P-FET. Across all SCE parameters, the simultaneous factors exhibit smaller variations. Notably, when considering their interaction through individual statistical summation (from Equation (1)), it becomes evident that all outcomes for various physical quantities are overestimated compared to simultaneous consideration of ALL factors. This underscores the importance of studying interactions comprehensively, considering ALL factors simultaneously rather than assessing them individually and summing the results. The complex interactions between the PVE and IPF factors result from their mutual annihilation and enhancement effects. Notably, a similar observation regarding IPF-induced band profile changes in the off-state was reported in our previous work for different MOSFETs [51,79]. Additionally, the impact of RDF is relatively smaller than that of other factors, owing to the strong screen effect of the GAA channel.

**Figure 6:**
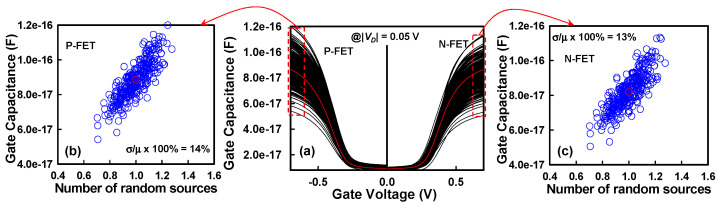
An AC curve operating at a signal frequency of 10 GHz is used as the basis for assessing the *C_G_*. (**a**) The *C_G_*-*V_G_* curves exhibit fluctuations due to the combined influence of PVE and IPF factors in CFET devices, affecting both N-FETs and P-FETs. (**b**) Variations in *C_G_* are shown with respect to the number of random sources for P-FETs in the on-state region. (**c**) *C_G_* fluctuations with respect to the number of random sources for N-FETs in the on-state region.

**Figure 7:**
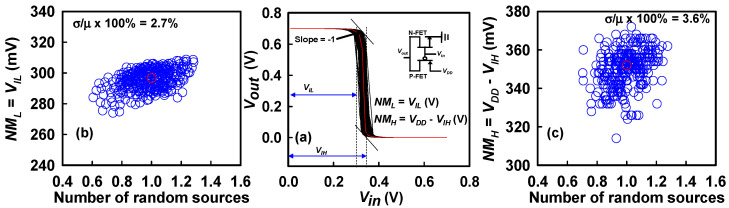
The voltage transfer characteristics of GAA Si NS CFET inverter circuits depict variations resulting from the concurrent impact of PVE and IPF factors. Notably, these fluctuations affect the assessment of the *NM* of the inverter, which is characterized by a slope of −1 *v/v* at two specific points on the voltage transfer curves, denoted as *V_IL_* and *V_IH_*. For added clarity, the definitions of both the *NM_L_* and the *NM_H_* are included in plot (**a**). (**b**,**c**) The plots illustrate fluctuations in *NM_L_* and *NM_H_* in relation to the number of random sources. It is noteworthy that *NM_L_* and *NM_H_* show an increasing trend as the number of random sources increases.

In this section, we focus on the analysis of the primary AC characteristic, namely the total *C_G_*, which plays a pivotal role in determining the dynamic switching behavior, signal delay, and overall frequency performance of GAA Si NS CFET devices. The total *C_G_* is directly extracted from the small-signal AC simulations performed in the strong inversion regime for both N-FETs and P-FETs, where the inversion carrier concentration is maximized, and the device operates in a quasi-saturation state. This extraction ensures that the measured capacitance reflects the intrinsic electrostatic coupling between the gate electrode and the inversion charge in the nanosheet channel. Moreover, recognizing the substantial influence of parasitic capacitances—originating from fringing fields, overlap capacitance between gate and source/drain, and fringe-to-substrate coupling—we have meticulously employed advanced calibration methodologies to incorporate these parasitic components into the calculated *C_G_* values. The methodology employs bias-dependent de-embedding techniques to accurately extract the intrinsic gate capacitance (C_G_) by isolating it from external parasitic contributions. Our AC simulations comprehensively account for all major parasitic components relevant to device-level AC performance. This includes intrinsic and fringing capacitances, as well as parasitic capacitances associated with the source/drain extensions, which are modeled based on realistic device geometries and calibrated doping profiles. Figure 6a depicts the fluctuations in *C_G_*−*V_G_* characteristics for N-FET and P-FET devices within the CFET structure, under the combined influence of PVE and IPF factors. The results clearly reveal that the synergistic interaction between PVE and IPF factors introduces significant fluctuations in the *C_G_*, reflecting sensitivity to nanoscale variations in channel dimensions, gate dielectric thickness, and material properties such as work function variations and interface trap densities. Figure 6b illustrates the extracted *C_G_* fluctuation trend for P-FETs as a function of the number of random sources. As the number of random sources increases, *C_G_* increases progressively. This increase can be attributed to enhanced electrostatic coupling arising from random local variations, such as effective gate length (*L_eff_*) reduction and nanosheet cross-sectional deformation. The RSD of *C_G_* for the P-FET is found to be 14%, indicating moderate susceptibility to stochastic process-induced variability. Similarly, Figure 6c illustrates the corresponding *C_G_* fluctuation trend for N-FETs. Consistent with P-FET behavior, *C_G_* in N-FETs also increases with the number of random sources, albeit with a slightly lower RSD variation of 13%. The near-similar magnitude of *C_G_* fluctuation between N-FETs and P-FETs suggests that both types of carriers (electrons and holes) are equally sensitive to variations in gate-to-channel coupling strength in GAA configurations. In GAA Si NS MOSFETs, the gate terminal fully encapsulates the NS channel from all sides, ensuring strong electrostatic control and minimized SCEs. However, this intimate wrapping also renders *C_G_* highly sensitive to PVE factors, such as variations in *W_NS_*, *T_NS_*, and gate dielectric uniformity. Thus, minor fluctuations in fabrication parameters can induce noticeable shifts in *C_G_*, impacting device delay and dynamic energy consumption. Transitioning to circuit-level implications, Figure 7a presents the VTC of a CFET-based inverter, highlighting the fluctuations induced by the simultaneous presence of PVE and IPF factors. Critical voltage points are extracted from the VTC: *V_IL_*, representing the maximum input voltage recognized as a logical ‘0’, and *V_IH_*, denoting the minimum input voltage identified as a logical ‘1’. These voltages correspond to the points on the VTC where the slope equals −1 V/V, a standard criterion for determining switching thresholds. Using *V_IL_* and *V_IH_*, the noise margins, namely *NM_L_* (low-level noise margin) and *NM_H_* (high-level noise margin), are calculated according to standard CMOS noise immunity definitions, as outlined in Figure 7a. The observed increase in *NM_L_* with rising N-FET threshold voltage may initially appear counterintuitive. This behavior can be understood by analyzing the inverter’s VTC. As the threshold voltage of the N-FET increases, the switching point of the inverter shifts toward higher input voltages, causing the transition from high to low output to occur later. This shift elevates the value of *V_IL_*, which is defined as the input voltage at which the slope of the VTC equals −1 during the low-to-high output transition. Since *NM_L_* = *V_IL_* − *V_OL_*, and *V_OL_* remains approximately constant, the increase in *V_IL_* directly contributes to a larger *NM_L_*. *NMs* are critical reliability metrics that quantify the maximum tolerable noise that a digital inverter can withstand without erroneous logic transitions during operation. Figure 7b,c, respectively, illustrate the extracted *NM_L_* and *NM_H_* variations as functions of the number of random sources. The results demonstrate that both *NM_L_* and *NM_H_* values increase as the number of random sources escalates. This trend arises due to the counteracting *Vth* shifts induced by variability: an increase in N-FET threshold voltage and a decrease in P-FET threshold voltage. These shifts lead to a corresponding increase in *V_IL_* and *V_IH_*, thereby improving *NMs* under variability-induced perturbations. The RSD variations for *NM_L_* and *NM_H_* are 2.7% and 3.6%, respectively, indicating relatively robust inverter performance against process-induced variations at the statistical level. Nevertheless, while increased *NMs* may seem advantageous, they also hint at possible increased switching delay due to higher thresholds, a tradeoff that must be carefully considered during circuit design for variability resilient CFET technologies. Thus, this comprehensive study reveals that in CFET-based circuits, the combined impacts of PVE and IPF factors manifest prominently not only at the device level through DC and AC characteristic degradations, but also propagate to circuit-level performance metrics, underlining the importance of co-optimization of device design, fabrication process control, and circuit architecture to mitigate the detrimental consequences of nanoscale variability.

**Figure 8:**
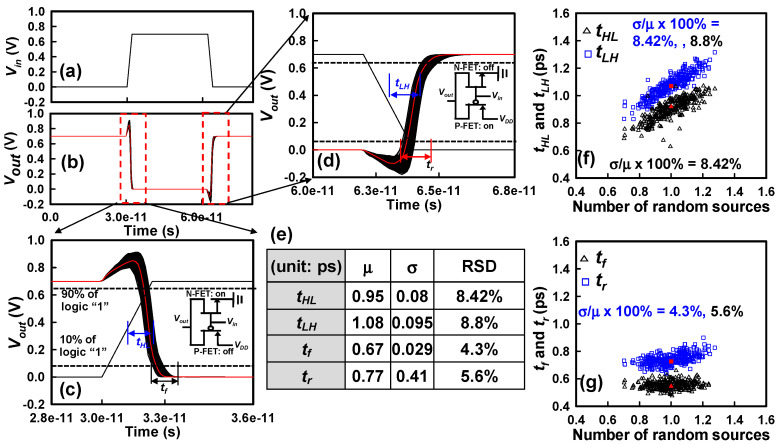
(**a**) The input signal applied to the CFET inverter. (**b**) Fluctuations observed in the output signals of the CFET inverter circuit for transient response, resulting from the combined influence of PVE and IPF factors. Zoomed-in plots of (**c**) the *t_HL_* and (**d**) the *t_LH_*. (**e**) A comprehensive list detailing the variations in timing. (**f**) Plots depicting the fluctuations in *t_HL_* and *t_LH_* concerning the number of combined PVE and IPF factors. (**g**) Graphical representations of the fluctuations in the *t_f_* and *t_r_* concerning the number of combined PVE and IPF factors.

Figure 8a,b illustrate the transient response characteristics of the GAA Si NS CFET inverter, providing detailed insights into the dynamic input–output behaviors under the combined influence of PVE and IPF factors. This transient behavior analysis is conducted under two fundamental switching conditions: (i) when the N-FET is active (on-state) and the P-FET is inactive (off-state), and (ii) when the P-FET is active, and the N-FET is inactive. These two distinct operational states are critical for comprehensively characterizing the CFET inverter’s dynamic response during different phases of logic transitions, specifically during the pull-down (logic ‘1’ to logic ‘0’) and pull-up (logic ‘0’ to logic ‘1’) events. To gain further resolution into the temporal characteristics, Figure 8c,d provide zoomed-in views of the output waveforms, illustrating the definitions and variations of key timing parameters: the *t_r_*, *t_f_*, *t_HL_*, and *t_LH_*. Here, *t_r_* represents the time interval for the output voltage (*V_out_*) to transition from 10% to 90% of the logic high level (*V_DD_*), while *t_f_* denotes the time required for *V_out_* to reduce from 90% to 10% of *V_DD_*. Similarly, *t_HL_* and *t_LH_* are defined as the time differences between the 50% transition points of the input and output signals during the falling and rising transitions, respectively. From a device physics standpoint, both *t_HL_* and *t_LH_* are strongly dependent on the *C_G_* and the drive currents of the corresponding switching FETs. Specifically, *t_HL_* is inversely proportional to the *I_on_* of the N-FET, while *t_LH_* is inversely proportional to the *I_on_* of the P-FET, indicating that higher drive currents facilitate faster switching transitions by enabling quicker charge/discharge of the load capacitance. Figure 8d highlights the transient behavior during the activation of the P-FET, revealing that the fluctuation of *t_LH_* is notably pronounced and predominantly attributed to the combined influence of PVE and IPF factors. A significant contributor to this increased variability is the presence of a parasitic leaky bottom channel in the P-FET structure, which degrades the effective drive strength and introduces additional timing uncertainty during the pull-up transition. Subsequently, Figure 8e summarizes the statistical metrics—mean, standard deviation, and RSD—for the timing parameter fluctuations observed in the CFET inverter under the combined influence of PVE and IPF factors. Figure 8f elaborates on the comparative fluctuations of *t_HL_* and *t_LH_*, highlighting that *t_HL_* is predominantly influenced by *Vth* fluctuations of the N-FET, while *t_LH_* is more significantly influenced by the *Vth* variability of the P-FET. It is important to note that the variation in *t_LH_* surpasses that of *t_HL_*, primarily because the P-FET exhibits a larger *Vth* fluctuation due to the weaker electrostatic control over the leaky bottom channel, a parasitic effect exacerbated by nanosheet thinning and gate stack non-uniformities. This inherent asymmetry in variability between pull-up and pull-down transitions underscores the necessity of careful device engineering to mitigate parasitic conduction paths in P-FETs for future CFET technologies. Figure 8g depicts the statistical fluctuations in *t_r_* and *t_f_*, revealing that the rise time fluctuation exceeds the fall time fluctuation. This observation is consistent with the fact that the drive strength of the P-FET (responsible for the pull-up transition) is inherently lower compared to that of the N-FET (responsible for the pull-down transition), a typical characteristic of CMOS and CFET technologies due to the disparity in hole and electron mobility. We now shift our focus to the impact of PVE and IPF factors on the various components of power consumption, namely the *P_static_*, *P_dyn_*, *P_sc_*, and *P_total_* [10,69]. The static power is quantified by the following formula:

(2) $$P_{static}=V_{DD}I_{leakage,}$$

where *I_leakage_* = *I_off_*_,_ N + *I_off_*, P, representing the cumulative leakage current flowing through the inverter during idle (non-switching) conditions, composed of the *I_off_* of the N-FET and P-FET. As *V_DD_* remains supplied continuously, *P_static_* persists irrespective of switching activity, making leakage control a critical challenge in ultra-scaled devices. The short-circuit power is expressed as follows:

(3) $$P_{sc}=f_{0\to 1}V_{DD}\int_{T}I_{sc}\left(\tau \right)d\tau ,$$

where *f*_0→1_ denotes the input transition frequency, and $I_{sc}\left(\tau \right)$ is the instantaneous short-circuit current that occurs when both the N-FET and P-FET conduct simultaneously during switching events, creating a transient direct path between *V_DD_* and ground.

The dynamic power, associated with charging and discharging of load capacitances during logic transitions, is given by the following:

(4) $$P_{dyn}=f_{0\to 1}{V_{DD}}^{2}C_{load},$$

where the load capacitance *C_load_* is predominantly determined by the sum of the gate capacitances of the N-FET and P-FET in their respective on-states.

(5) $$C_{load}=C_{G,N}+C_{G,P}.$$

In advanced GAA Si NS CFETs, the superior electrostatic gate control effectively reduces parasitic capacitances and enhances switching efficiency, thus contributing to minimized dynamic power consumption compared to planar or FinFET counterparts.

The total power consumption of the CFET inverter is therefore:

(6) $$P_{total}=P_{static}+P_{SC}+P_{dyn}.$$

**Figure 9:**
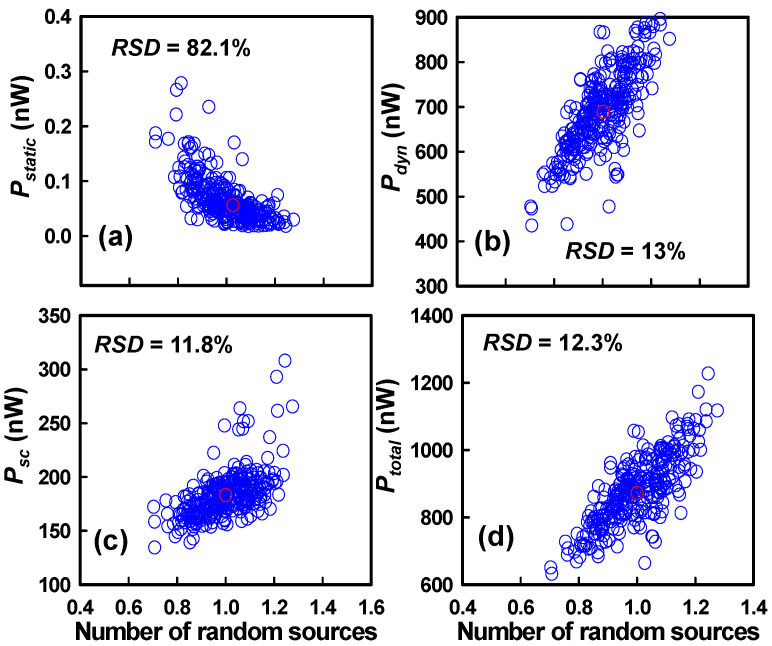
(**a**–**d**) Variations in *P_static_*, *P_dyn_*, *P_sc_*, and *P_total_* of the GAA Si NS CFET-based inverter circuit as a function of the number of random sources originating from the combined impact of PVE and IPF factors. These fluctuations arise from stochastic variations in key device parameters such as *Vth*, *L_eff_*, and mobility, which in turn modulate leakage currents, switching dynamics, and transient behavior. As a result, each component of power consumption exhibits distinct sensitivity to the underlying variability mechanisms, emphasizing the critical role of variability-aware modeling in low-power and high-reliability circuit design at advanced technology nodes.

Figure 9 comprehensively examines the impact of PVE and IPF factors on these power components. Specifically, Figure 9a–d depict the statistical distribution and fluctuation trends of *P_static_*, *P_dyn_*, *P_sc_*, and *P_total_*, respectively, as a function of increasing numbers of random sources. The simulation results reveal that three power components exhibit an upward trend with the increasing number of random sources. However, the *P_static_* exhibits an opposite trend compared to the other power components, showing a decreasing behavior with the increasing number of random sources. As defined in Equation (2), *P_static_* is calculated based on the *I_leakage_* = *I_off_*_,_ N + *I_off_*, P of both the N-FET and P-FET in the off-state. Therefore, its trend closely follows the behavior of *I_off_* as illustrated in Figure 4d,g. Among them, *P_static_* demonstrates the highest sensitivity, with an RSD of approximately 82.1%, which far exceeds the RSD values observed for *P_dyn_* (13.0%), *P_sc_* (11.8%), and *P_total_* (12.3%). The *P_static_* exhibits the highest RSD among all power components; however, its absolute magnitude is substantially smaller than *P_total_*; consequently, despite its high RSD, the contribution of *P_static_* to the overall power variation remains negligible. The pronounced variability of *P_static_* can be directly attributed to its linear dependence on *I_leakage_*, which itself is highly sensitive to *Vth* fluctuations. As shown in Figure 4d,g, the divergent trends in *I_off_*, N and *I_off_*, P under variability lead to an overall amplification of static power fluctuations. Interestingly, while *P_dyn_*, *P_sc_*, and *P_total_* follow relatively steady trends, the anomalous behavior of *P_static_* underscores the critical need to control leakage mechanisms, such as gate-induced drain leakage (*GIDL*) and subthreshold leakage, particularly in P-FETs, where parasitic channels exacerbate the variability. In summary, the markedly higher RSD observed for *P_static_* highlights the heightened vulnerability of leakage-driven power dissipation to PVE and IPF factors, emphasizing the importance of adopting variability-aware design and optimization strategies in next-generation GAA Si NS CFET technologies.

**Figure 10:**
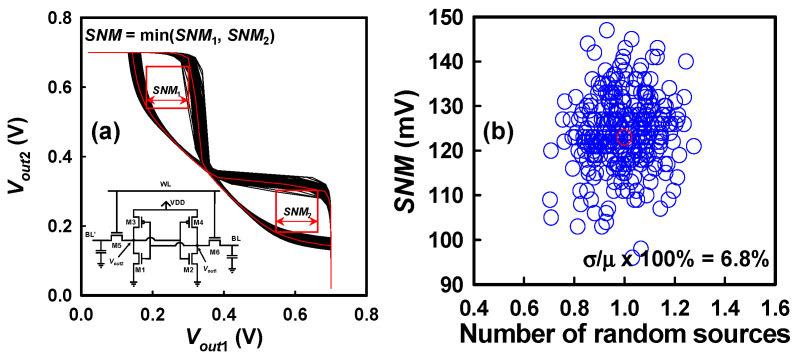
(**a**) Static characteristic curves of the 6T-SRAM cell showing fluctuations caused by the combined influence of PVE and IPF factors. The inset is a 6T CFET SRAM circuit. (**b**) Fluctuations in the *SNM* concerning the number of combined PVE and IPF factors.

Extensive research efforts have been devoted to the development and optimization of diverse SRAM cell topologies [80], with a particular emphasis on variation-aware design methodologies and statistical variability modeling frameworks to address the critical challenges posed by device-level randomness at advanced technology nodes [81]. As technology scaling continues into the sub 5 nm regime, random variations—including PVE and IPF factors—have become increasingly significant, necessitating the exploration of SRAM architectures capable of maintaining performance, stability, and energy efficiency under variability stress [82]. Numerous SRAM cell designs have been proposed to achieve key design targets, such as ultra-low standby and dynamic power consumption, high-speed operation, and enhanced read/write stability margins, often incorporating techniques such as device sizing optimization, assist circuits, and variability mitigation strategies tailored to specific process technologies and device architectures, including FinFETs and GAA NS MOSFETs [80,81]. Among the various architectures, the conventional 6T SRAM cell has remained the industry standard due to its relatively simple fabrication process, compact area efficiency, and the capacity to achieve full logic-level output voltage swings during read and write operations. The classical 6T SRAM structure is composed of two cross-coupled CMOS inverters forming a bistable latch for data storage, complemented by two NMOS access transistors that enable read and write access by selectively coupling the internal storage nodes to the bit lines, as schematically shown in the inset of Figure 10a. However, the robustness of the 6T SRAM is heavily influenced by transistor mismatch, where random fluctuations in *Vth*, effective channel length (*L_eff_*), carrier mobility (*μ_eff_*), and PVE parameters across the paired inverters can induce asymmetrical switching behavior. This mismatch leads to degradation in both read and write noise margins, increases the probability of read–disturb or write–failure events, and ultimately impacts the overall functional yield of large-scale SRAM arrays. In this context, a comprehensive understanding of the combined influence of PVE and IPF factors on the *SNM*—a critical metric for SRAM cell stability—is essential for enabling robust, variability-resilient design strategies. To systematically investigate these effects, we examine the combined impact of PVE and IPF factors on the *SNM* of a 6T SRAM cell based on GAA Si NS CFET technology. Figure 10a illustrates the static VTCs of the SRAM cell under the influence of these variability sources. The VTCs, obtained by sweeping the internal storage node voltages, clearly exhibit noticeable distortions and shifts when subjected to random variations, reflecting significant perturbations in the voltage thresholds and inverter gain, and thus indicating substantial instability in the cell’s bistable operation. Furthermore, Figure 10b illustrates the corresponding statistical distribution of *SNM* values as a function of the number of concurrently active random variation sources included in the simulation framework. A strong anti-correlation is observed between the *SNM* and the fluctuations in the read current (*I_read_*) through the access transistor. This inverse relationship can be explained by considering that an increased pull-down strength (or enhanced pass-gate drive current) during the read operation elevates the internal storage node voltage, thus narrowing the metastability window and reducing the stability margin of the cell. Such a phenomenon increases the susceptibility of the cell to read–disturb failures, wherein a read operation inadvertently flips the stored data, especially under marginal *SNM* conditions. Quantitatively, the *SNM* variability exhibits an RSD of approximately 6.8%, demonstrating that the 6T SRAM cell is highly sensitive to the superposition of device-level random variability sources. This finding underscores the urgent need for variability-tolerant design approaches, including upsizing of pull-down transistors, adaptive body biasing, or the adoption of alternative topologies, such as 8T or 10T cells in variability-prone applications. Particularly for technology nodes incorporating GAA NS devices and CFET architectures, where device electrostatics and SCEs are significantly enhanced but variability remains a critical challenge, integrating robust statistical design methodologies becomes indispensable for ensuring reliable SRAM operation and acceptable yield in advanced integrated circuits.

## 4. Conclusions

In summary, our investigation examined the combined impact of PVE, WKF, and RDF on vertically stacked GAA Si NS CFETs. Among the various factors investigated within PVE, the *T_NS_*, *W_NS_*, and *L_G_* emerge as pivotal contributors to fluctuations in characteristics. In essence, it is plausible to mitigate the impact of PVE; however, careful consideration must be given to *T_NS_*, *W_NS_*, and *L_G_* during the GAA Si NS CFET process. The effects of WKF and RDF in IPF, the SCE parameters are effectively suppressed for both N- and P-FETs of CFETs, owing to increased gate area of the work function metal and superior channel control. The use of amorphous type MGs in WKF results in a reduction in the characteristic fluctuation. The key findings of our study reveal that PVE and IPF factors exert a more pronounced effect on P-FETs than N-FETs. Specifically, we observed a substantial 131% variation in *I_off_* of P-FET under the influence of these combined random sources. Owing to the statistically independent and identically distributed random sources, the individual statistical summation of RSD for each physical quantity results in an overestimation compared to the cumulative effect of considering all sources simultaneously. Although the nominal value of *P_static_* is comparatively marginal when contrasted with the magnitudes of *P_dyn_* and *P_sc_*, it exhibits the most significant variation, reaching up to 82% during the off-state operation of CFETs. In addition to this, we also explored the variation of *C_G_* in GAA Si NS CFETs under the combined influence of these factors. It is important for us to note that we did not consider the coupling capacitance for N-/P-FETs in this study, which may have implications for circuit speed and power consumption. Our future research will concentrate on investigating the effect of coupling capacitance in high-speed circuits. These results provide valuable insights for characterizing and optimizing PVE and IPF factors in CFET fabrication and CFET-based circuit design.

## Figures and Tables

**Table 1:** Adopted scaled device (*L_G_* = 16 nm) parameters for the investigated vertically stacked GAA Si NS CFET [10], along with the extracted SCE metrics for the nominal device.

**Device Parameter**	**Value**	
Channel length (nm) (*L_G_*)	16	
Channel doping (cm^−3^)	6 × 10^16^	
S/D extension (nm)	5	
S/D length (nm)	12	
Effective oxide thickness (nm) (*EOT*)	0.66	
Nanosheet thickness (nm) (*T_NS_*)	5	
Nanosheet width (nm) (*W_NS_*)	16	
Work function (eV)	4.45, 4.80	
S/D doping (cm^−3^)	1 × 10^20^	
S/D extension doping (cm^−3^)	5 × 10^18^	
**The achieved characteristics**	**N-FET**	**P-FET**
Threshold voltage (*Vth*) (mV)	260	−260
Off-state current (*I_off_*) (A)	3.5 × 10^−11^	4.11 × 10^−11^
On-state current (*I_on_*) (A)	3.3 × 10^−4^	2 × 10^−4^
Subthreshold slope (*SS*) (mV/dec)	62	72
Drain-induced barrier lowering (*DIBL*) (mV/V)	30	49

**Table 2:** Comparison of SCE parameter variations due to individual random sources (PVE, WKF, RDF) and simultaneously combined factors (ALL), highlighting the correlation between simultaneous and statistical summation methods for individual factors.

Parameter	Device	*T_NS_*	*W_NS_*	*L_G_*	*WKF*	*RDF*	*RSD_ALL_*	*RSD_SUM_*
RSD of *I_on_* (%)	N-FET	9.35	6.4	1.6	3.9	1.3	10.8	12.2
	P-FET	14.4	10.4	3.27	2.3	2	13	18.3
RSD of *I_off_* (%)	N-FET	50.4	30.1	37.5	60.5	10.1	65	92.75
	P-FET	123	45.3	57.4	48.4	21.7	131	152.6
RSD of *Vth* (%)	N-FET	3.3	3.1	2.4	4.9	0.8	4.9	7.2
	P-FET	4.6	3.8	3.5	3.4	1.1	6.1	7.8
RSD of *DIBL* (%)	N-FET	14.4	12.7	5.6	16.6	5.82	17.7	26.7
	P-FET	16.5	13.5	8.2	9.8	5.89	20.7	25.6
RSD of *SS* (%)	N-FET	1.05	0.95	1.0	23.7	26.4	1.1	35.6
	P-FET	4.8	2.8	1.94	42.6	21.8	4.5	48.2
RSD of *g_m_* (%)	N-FET	7.5	9.76	3.4	45.2	11	9.3	48.3
	P-FET	16.9	10.2	5.43	42.6	16.2	78	50

## Data Availability

Data are contained within the article.
